# Statins Induce Actin Cytoskeleton Disassembly and an Apoptosis-Like Process in *Acanthamoeba* spp.

**DOI:** 10.3390/antibiotics11020280

**Published:** 2022-02-21

**Authors:** Rubén L. Rodríguez-Expósito, Ines Sifaoui, María Reyes-Batlle, Sutherland K. Maciver, José E. Piñero, Jacob Lorenzo-Morales

**Affiliations:** 1 Instituto Universitario de Enfermedades Tropicales y Salud Pública de Canarias, Universidad de la Laguna (ULL), 38206 Tenerife, Spain; rrodrige@ull.edu.es (R.L.R.-E.); isifaoui@ull.edu.es (I.S.); mreyesba@ull.edu.es (M.R.-B.); 2 Departamento de Obstetricia, Ginecología, Pediatría, Medicina Preventiva y Salud Pública, Toxicología, Medicina Legal y Forense y Parasitología, Universidad de la Laguna (ULL), La Laguna, Tenerife, 38203 Islas Canarias, Spain; 3 Red de Investigación Cooperativa en Enfermedades Tropicales (RICET), 28029 Madrid, Spain; 4 Centre for Integrative Physiology, Biomedical Sciences, Edinburgh Medical School, University of Edinburgh, Edinburgh EH8 9AG, UK; sutherland.maciver@ed.ac.uk; 5 CIBER de Enfermedades Infecciosas (CIBERINFEC), Instituto de Salud Carlos III, 28029 Madrid, Spain

**Keywords:** *Acanthamoeba*, cerivastatin, pitavastatin, rosuvastatin, phalloidin, PCD

## Abstract

*Acanthamoeba* is a ubiquitous opportunistic protozoan pathogen that is known to cause blinding keratitis and rare, but usually fatal, granulomatous encephalitis. The difficulty in treating infections and the toxicity issues of the current treatments emphasize the need to use alternative agents with amoebicidal activity. The aim of this study was to evaluate the in vitro antiamoebic activity of three third-generation statins—cerivastatin, pitavastatin and rosuvastatin—against both cysts and trophozoites of the following four strains of *Acanthamoeba: A. castellanii* Neff, *A. polyphaga, A. griffini* and *A. quina*. Furthermore, programmed cell death (PCD) induction traits were evaluated by measuring chromatin condensation, damages at the mitochondrial level, production of reactive oxygen species (ROS) and the distribution of actin cytoskeleton fibers. *Acanthamoeba castellanii* Neff was the strain most sensitive to all the statins, where cerivastatin showed the lowest amoebicidal activity for both trophozoite and cyst forms (0.114 ± 0.050 and 0.704 ± 0.129 µM, respectively). All the statins were able to cause DNA condensation, collapse in the mitochondrial membrane potential and a reduction in ATP level production, and disorganization of the total actin fibers in the cytoskeleton of all the evaluated *Acanthamoeba* strains. Our results showed that the tested statins were able to induce PCD compatible events in the treated amoebae, including chromatin condensation, collapse in the mitochondrial potential and ATP levels, cytoskeleton disassembly and ROS generation.

## 1. Introduction

*Acanthamoeba* is a genus of pathogenic, free-living amoebae that can cause several opportunistic infections in humans, namely, *Acanthamoeba* keratitis (AK), granulomatous amoebic encephalitis (GAE) and disseminated infections (mostly cutaneous and nasopharyngeal) [1]. Based on rRNA gene sequence analyses, 23 different genotypes have been reported to date (T1 to T23), with T4 being the most abundant genotype in both clinical and environmental samples. *Acanthamoeba* are mixotrophic and can encyst under unfavorable conditions [2,3]. Currently, the treatment of *Acanthamoeba* infections remains an issue, due to the presence of cyst forms and to the variability in drug efficacy among different genotypes, in addition to the long periods of drug administration and drug toxicity. Hence, there is an urgent need to validate new amoebicidal molecules that target essential biosynthesis pathways in *Acanthamoeba*. Ergosterol and 7-dehyrostigmasterol have been reported as the major sterols on the membrane of *Acanthamoeba,* for both cyst and trophozoite stages [4]. Being vital to *Acanthamoeba*, the ergosterol biosynthesis pathway could be used as a potential drug target [5,6]. The 3-hydroxy-3-methylglutaryl coenzyme A reductase (HMG-CoA) enzyme catalyzes the synthesis of ergosterol in fungi, plant, and free-living amoeba; consequently, inhibiting or blocking this enzyme should induce irreversible damage in the amoebic membrane [5].

Statins are cholesterol-lowering drugs that are primarily used for hypercholesterolemia therapy [7]. These molecules act as competitive inhibitors of 3-hydroxy-3-methylglutaryl coenzyme A (HMG-CoA) reductase [1]. Three generations of statins were developed, based on their efficacy to lower the plasma low-density lipoprotein cholesterol (LDL-C) concentration. The first-generation statins include lovastatin, pravastatin and fluvastatin, while the second generation includes atorvastatin, and rosuvastatin, pitavastatin and cerivastatin constitute the third-generation statins [8].

In previous studies, we have already confirmed the amoebicidal activity of many statins, namely, fluvastatin and pravastatin, which are first-generation statins, and atorvastatin and simvastatin, which are second-generation statins [5]. Martin-Navarro et al. (2015) have confirmed that statins can induce irreversible cell membrane damage, as well as apoptotic-like features in *A. castellanii* Neff, including chromatin condensation [1]. In addition, Hahn et al. (2020) have recently reported that the amoebicidal activity of pitavastatin against *A. castellanii* Neff was 10 times higher than that of rosuvastatin [9]. In the present work, the amoebicidal activity of three different third-generation statins against the cyst and trophozoite stages of four *Acanthamoeba* strains was evaluated. In addition, their effect on actin distribution and the induction of different apoptosis-like features were studied, including chromatin condensation and mitochondrial damage.

## 2. Results

### 2.1. In Vitro Activity of Statin against Acanthamoeba spp.

In the present study, three different third-generation statins were evaluated to identify their activities against four *Acanthamoeba* strains. All the IC_50_ values are summarized in Table 1. The ANOVA analysis revealed that both trophocidal and cysticidal activities were significantly dependent on the strain and statin used, with *p* < 0.0001.

Indeed, for both cysticidal and trophocidal activities, *Acanthamoeba castellanii* Neff was the most sensitive strain, while *Acanthamoeba polyphaga* was the most resistant. As for the type of statin used, rosuvastatin was less active against both cyst and trophozoite forms of all the tested strains. As for cerivastatin and pitavastatin, their amoebicidal activity was not statistically significant, except for the cyst stage of *Acanthamoeba polyphaga,* where cerivastatin was more active than pitavastatin.

### 2.2. Statins Induced Actin Cytoskeleton Disassembly

**Figure 1:**
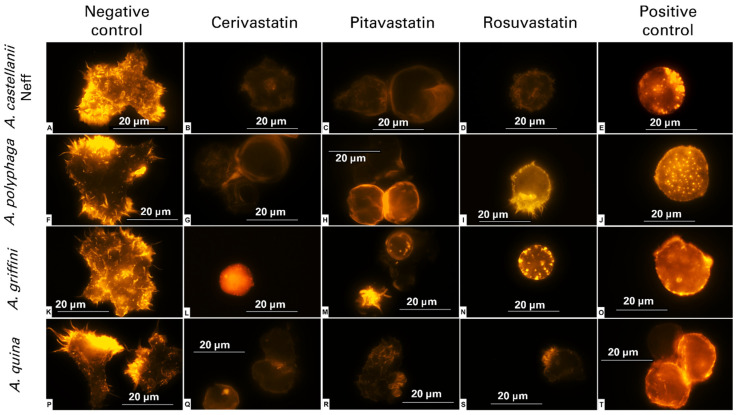
Trophozoites of *Acanthamoeba castellanii* Neff (**A**–**E**), A. polyphaga (**F**–**J**), A. griffini (**K**–**O**) and A. quina (**P**–**T**) incubated with the IC_90_ of cerivastatin (**B**,**G**,**L**,**Q**), pitavastatin (**C**,**H**,**M**,**R**) and rosuvastatin (**D**,**I**,**N**,**S**) for 24 h. The positive control was processed by treating amoebae with methyl-β-cyclodextrin (**E**,**J**,**O**,**T**). The phalloidin-TRITC dye stained the actin cytoskeleton, showing a disorganization effect in the positive control and in the amoebae treated with statins, and emitted a lower orange fluorescence than the non-treated cells (negative control: **A**,**F**,**K**,**P**). All images were captured using the EVOS FL Cell Imaging System EVOS™ M5000 (100× magnification). Images shown are representative of the cell populations observed in each experimental condition.

Actin networks and the organization of cytoskeletal structures are indispensable elements of the cellular morphology, adhesion, motility and pathogenicity of adherent cells, namely, *Acanthamoeba* [10]. Thus, we inspected the eventual effect of statins on the cytoskeleton and the actin’s network, using specific actin staining. After 24 h of incubation with the statins at IC_90_, (Table 2) the rounded cells emitted less intense fluorescence than the negative control (Figure 1A,F,K,P), reflecting complete disorganization of the actin fibers and total loss of the cytoskeletal structure (Figure 1).

### 2.3. Statin Treatments Induce Compatible Events Related to Programmed Cell Death of Amoebae

**Figure 2:**
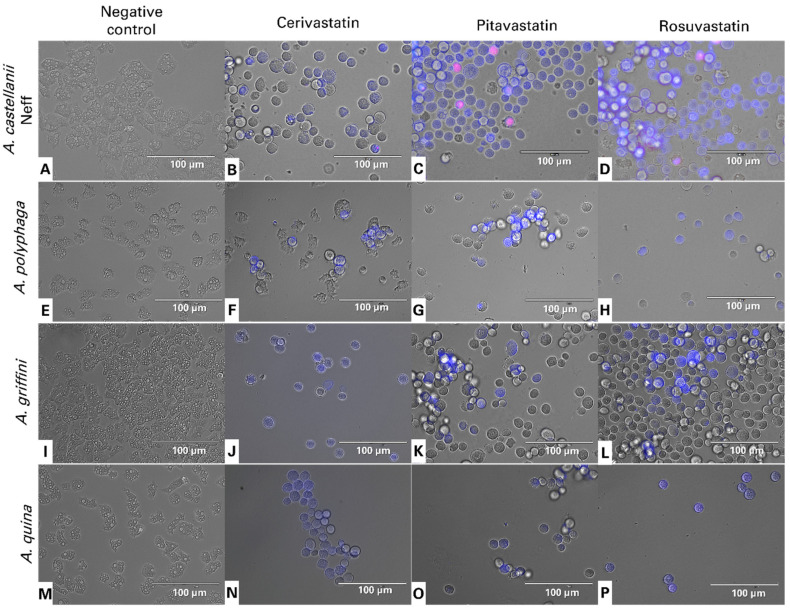
Images of *Acanthamoeba castellanii* Neff (**A**–**D**), *A. polyphaga* (**E**–**H**), *A. griffini* (**I**–**L**) and *A. quina* (**M**–**P**) showing the effect of IC_90_ of cerivastatin (**B**,**F**,**J**,**N**), pitavastatin (**C**,**G**,**K**,**O**) and rosuvastatin (**D**,**H**,**L**,**P**), relative to the negative control (non-treated amoebae) (**A**,**E**,**I**,**M**), after 24 h of incubation in vitro. Hoechst stains treated trophozoites nuclei in bright blue, showing chromatin condensation, while red fluorescence corresponds to the propidium iodide dye, which stains the DNA of dead cells. All images were captured using an EVOS FL Cell Imaging System EVOS™ M5000 (40× magnification). Images shown are representative of the cell populations observed in each experimental condition.

The lipid-lowering drugs, statins, were reported to induce apoptosis in a variety of tumor cells [11], as well as free-living amoeba, such as *Acanthamoeba* [1], *Naegleria fowleri* [12] and *Trypanosoma* [13] among others. From Figure 2, we can distinguish the following three groups: non-stained cells as healthy cells; cells undergoing an early apoptosis process, with bright blue stained nuclei; cells in a late apoptotic process, stained blue and red.

### 2.4. Statins Decreased ATP Levels during the Time of Incubation

**Figure 3:**
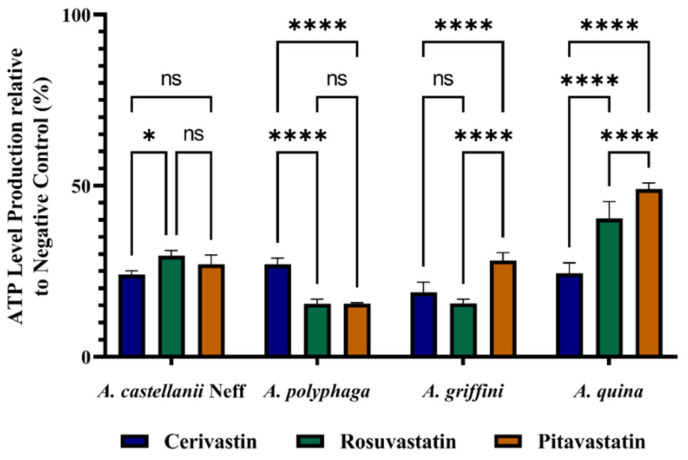
Effect of IC_90_ of statins on the ATP production of *Acanthamoeba castellanii* Neff, *A. polyphaga*, *A. griffini* and *A. quina* trophozoites, using CellTiter-Glo^®^ luminescent cell viability assay. Results are represented as percentages relative to the negative control. Differences between the values were assessed using one-way analysis of variance (ANOVA). Data are presented as means ± SD (N = 3); ns, non-significant. * *p* < 0.05 and **** *p* < 0.0001 are significance differences when comparing the different mean values.

We further evaluated the effect of the statins on the total ATP levels produced in the treated cells, relative to the control. We found that all the statins could reduce ATP production by at least 50%. The reductions were affected by both the type of strain and molecules used. For *Acanthamoeba quina,* the ATP produced by the different statins was statistically significant, with a higher decrease induced by cerivastatin. However, in *Acanthamoeba castellanii* Neff, the difference between the three molecules was non to slightly significant (Figure 3).

#### 2.4.1. Statins Induced the Collapse of Mitochondrial Membrane Potential (ΔΨm)

**Figure 4:**
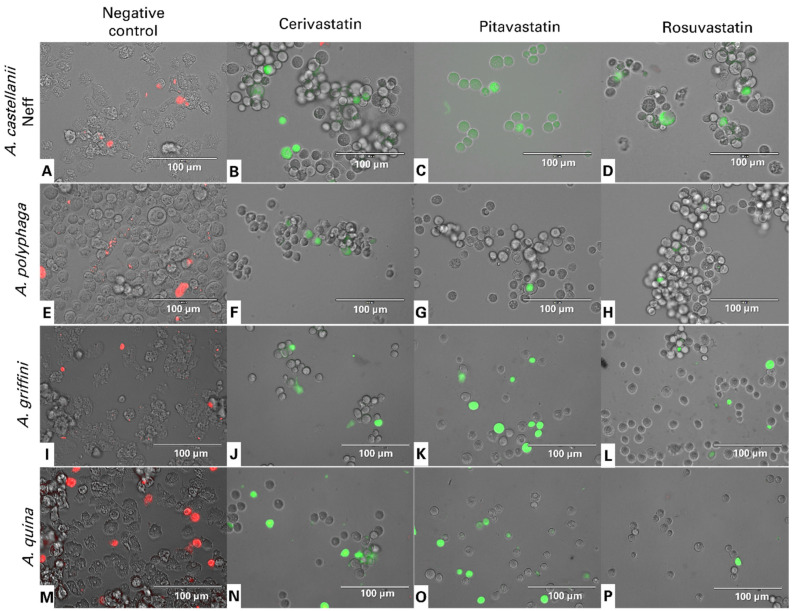
JC-1 dye showing the effect of IC_90_ of cerivastatin (**B**,**F**,**J**,**N**), pitavastatin (**C**,**G**,**K**,**O**) and rosuvastatin (**D**,**H**,**L**,**P**) in trophozoites of *Acanthamoeba castellanii* Neff (**A**–**D**), *A. polyphaga* (**E**–**H**), *A. griffini* (**I**–**L**) and *A. quina* (**M**–**P**) for 24 h of incubation. JC-1 dye remained in its monomeric form inside the treated cells, emitting green fluorescence, suggesting that the statins could induce depolarization of the mitochondrial potential. In healthy trophozoites, JC-1 dye accumulated in aggregates inside the mitochondria, emitting red fluorescence (**A**,**E**,**I**,**M**). All images were captured using an EVOS FL Cell Imaging System EVOS™ M5000 (40× magnification). Images shown are representative of the cell populations observed in each experimental condition.

The mitochondrial membrane potential, (ΔψM), is an indispensable element for good mitochondrial function. JC-1 is a permeable, lipophilic, cationic dye that can selectively stain mitochondria and reversibly change color from its green monomer form to red, as the membrane potential increases, for its dimeric form. Figure 4 shows the JC-1 dye emitting green fluorescence, showing that all the statins can induce depolarization of the mitochondrial potential. Indeed, JC-1 remained in the cytoplasm in its monomeric form. In healthy cells, the high mitochondria membrane potential allows the dye to accumulate and aggregate in the mitochondria and emit a red fluorescence signal (Figure 4A,E,I,M).

#### 2.4.2. Statins Increase Reactive Oxygen Species (ROS) Levels in Acanthamoeba

**Figure 5:**
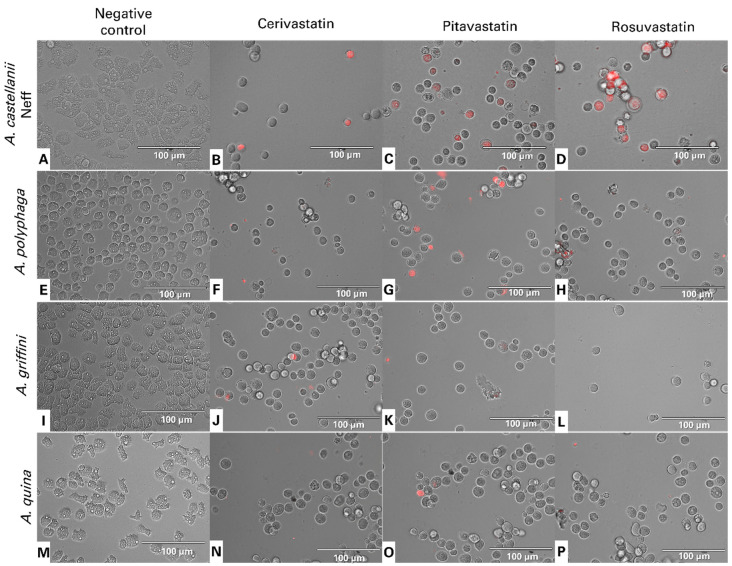
Detection of reactive oxygen species (ROS) in trophozoites of *Acanthamoeba castellanii* Neff (**A**–**D**), *A. polyphaga* (**E**–**H**), *A. griffini* (**I**–**L**) and *A. quina* (**M**–**P**), treated with the IC_90_ of cerivastatin (**B**,**F**,**J**,**N**), pitavastatin (**C**,**G**,**K**,**O**) and rosuvastatin (**D**,**H**,**L**,**P**) for 24 h, and comparing with the negative control (**A**,**E**,**I**,**M**). CellROX™ Deep Red Reagent emitted bright red fluorescence in the presence of ROS. All images were captured using an EVOS FL Cell Imaging System EVOS™ M5000 (40× magnification). Images shown are representative of the cell populations observed in each experimental condition.

In order to evaluate the effect of the statins on the cell’s oxidative state, staining, using CellROX Deep RedTM, was conducted. Figure 5 shows that the treatment of *Acanthamoeba* spp. with different statins noticeably enhanced the red fluorescence, particularly in *Acanthamoeba castellanii* Neff, reflecting the increase in ROS intracellular production.

## 3. Discussion

Statins are widely used in hypercholesterolemia treatment, due to their great inhibition of 3-hydroxy-3-methylglutaryl coenzyme A (HMG-CoA) reductase [14]. This enzyme catalyzes the reduction of 3-hydroxy-3-methylglutaryl-CoA (HMG-CoA) to mevalonate, which is considered a mandatory step in cholesterol and ergosterol biosynthesis [15]. In addition, several reports have recently shown that statins can affect cytoskeleton distribution, resulting in a loss of cell surface, motility and adhesion. Kuipers et al. (2006) have reported that simvastatin can affect the shape and adhesive capacity of microglia cells by altering their actin network [14]. In our previous work, we identified the gene encoding for *Acanthamoeba* HMG-CoA reductase, and we reported the use of statins as amoebicidal drugs via the inhibition of the last enzyme. Ergosterol and 7-dehyrostigmasterol constitute the main sterols of *Acanthamoeba*’s membrane. Therefore, blocking their synthesis normally led to the membrane architecture of *Acanthamoeba* becoming defective [1,5].

Among the three tested statins, the amoebicidal activity of pitavastatin and rosuvastatin has been reported in *A. castellanii*. The authors found that pitavastatin was more active than rosuvastatin, and it could inhibit the following different *Acanthamoeba* strains: CDC:V240 and MEEI 0184 [9]. In concordance, in the present work, cerivastatin and pitavastatin were more active than rosuvastatin against both stages of the four *Acanthamoeba* strains tested.

Phalloidin staining was processed to visualize the effect of the statins on *Acanthamoeba*’s actin network. Phalloidin is a fungal toxin that specifically binds to F-actin, resulting in the stabilization of actin filaments. We could visualize that the cells treated with statins emitted lower fluorescence than the negative control, demonstrating that the present drugs can induce actin stress fiber distribution. Phalloidin staining in the negative control revealed the presence of regular actin filaments and a highly organized dense network. In contrast, most of the treated cells lost their membrane architecture and showed the presence of cytoskeleton disorganization. The effect of the statins on the actin cytoskeletons was similar to that elicited by methyl-β-cyclodextrin treatment, which is known to sequester membrane sterols, supporting the contention that statins act on actin cytoskeletons by lowering sterols. Effectively, statins could affect the cytoskeleton of *Acanthamoeba* spp., and this would result in rounded cells, without a defined shape. Similar morphological features were observed in a cardiac fibroblast by Copaja et al. (2012). The authors reported that cells treated with simvastatin presented smaller sizes, rounder shapes and complete disruptions to their actin networks [16]. In another report, Sokalska et al. (2010) proposed a mechanism of action of simvastatin on the cytoskeleton of endometrial stromal cells via the reduction of geranylgeranyl diphosphate and, hence, the decrease in protein geranylgeranylation, namely, in Rho, Rac, and Cdc42. In fact, those proteins regulate the organization of actin cytoskeletons, and could constitute a good target to inhibit *Acanthamoeba* cells by preventing their adhesion and damaging their cytoskeleton [17].

In addition to their clinical use to lower serum cholesterol levels, statins have been reported to induce apoptosis in several experimental models, namely, tumors, such as C6 glioma cells, acute myeloid leukemia and human lymphocytes. Tsubaki et al. (2015) reported that simvastatin and fluvastatin can inhibit metastatic mouse melanoma growth via the abolition of cell motility and cycle progress keys, such as metalloproteinases (MMPs), very late antigens (VLAs) and Rho prenylation [18]. As for pitavastatin, it was described to induce apoptosis in oral cancer cells via the activation of caspase 3/9 [19].

Regarding protists, it has been demonstrated that atorvastatin, and other drugs that interfere with the sterol biosynthetic pathway, induce PCD in trypanosomatids [13]. In *Acanthamoeba,* we demonstrated, in previous work, that atorvastatin, fluvastatin, simvastatin and voriconazole induce cellular DNA fragmentation and chromatin condensation [1]. Furthermore, 24 h of incubation with those statins induces apoptosis in *Acanthamoeba castellanii* Neff via caspase 3. In the present work, the three tested statins could induce oxidative stress in *Acanthamoeba* spp. by increasing the production of ROS. Further experiments evaluating the effect of ROS accumulation on proapoptotic markers would be interesting.

## 4. Materials and Methods

### 4.1. Chemicals

In the present study, three different statins were evaluated for their amoebicidal activity, namely, cerivastatin, rosuvastatin and pitavastatin. All molecules were purchased from Cayman Chemicals (Vitro SA, Madrid, Spain). As a positive control, chlorhexidine was used. Stock solutions were prepared in dimethyl sulfoxide (DMSO) and maintained at −20 °C until required. In the whole experiment, a maximum of 0.1% of DMSO was used.

### 4.2. Acanthamoeba Strains

An amoebicidal effect was observed against the following four strains of *Acanthamoeba*: *Acanthamoeba castellanii* Neff, genotype T4 (ATCC 30010) strain from the American Type Culture Collection; *Acanthamoeba griffini*, genotype T3 obtained in previous studies (14); *Acanthamoeba polyphaga*, genotype T4 (ATCC 30461); *Acanthamoeba quina*, genotype T4 (ATCC 50241). Those strains were grown axenically at 26 °C for 3 days in PYG medium (0.75% (*w*/*v*) proteose peptone, 0.75% (*w*/*v*) yeast extract and 1.5% (*w*/*v*) glucose), containing 40 μg gentamicin mL^−1^ (Biochrom AG, Cultek, Granollers, Barcelona, Spain), before being used.

### 4.3. In Vitro Effect against the Trophozoite Stage of Acanthamoeba

The anti-*Acanthamoeba* activity of the tested molecules was determined by alamarBlue^®^ Reagent assay, as previously described [20]. Briefly, *Acanthamoeba* strains were seeded in triplicate on a 96-well microtiter plate with 50 μL from a stock solution of 5 × 10^4^ cells/mL. Amoebae were allowed to adhere for 15 min and 50 μL of serial dilution series of the drug was added. Finally, the alamarBlue^®^ Reagent was added into each well at an amount equal to 10% of the medium volume (Life Technologies, Madrid, Spain). The plates were then incubated for 96 h at 26 °C with slight agitation and the emitted fluorescence was measured with an EnSpire^®^ Multimode Plate Reader (Perkin Elmer, Madrid, Spain) at 570/585 nm.

### 4.4. In Vitro Effect against the Cyst Stage of Acanthamoeba spp.

Preparation of mature cysts.

Briefly, trophozoites were transferred from PYG medium-based cultures (trophozoite medium) to Neff’s encystment medium (NEM; 0.1 M KCl, 8 mM MgSO_4_·7H_2_O, 0.4 mM CaCl_2_·2H_2_O, 1 mM NaHCO_3_, 20 mM ammediol [2-amino-2-methyl-1,3-propanediol; Sigma Aldrich Chemistry Ltd., Madrid, Spain], pH 8.8, at 25 °C) and were cultured in this medium with gentle shaking for two weeks in order to obtain mature cysts. After that, mature cysts were harvested and washed twice using PYG medium [21].

The cysticidal activity was determined by the alamarBlue^®^ method at 144 h and confirmed visually by inverted microscopy. The in vitro susceptibility assay was performed in sterile 96-well microtiter plates (Corning™, New York, NY, USA). First, a serial dilution of the tested molecules was made in PYG. Then, 50 µL of mature cysts at a concentration of 5 × 10^4^/mL were added to all the wells. The plates were examined with inverted microscopy, and after 7 days of incubation, the plates were centrifuged at 800× *g* for 10 min and the medium was replaced with new PYG. Finally, 10 μL of the alamarBlue^®^ Reagent (Biosource, Europe, Nivelles, Belgium) was placed into each well, and the plates were then incubated for 144 h at 26 °C with slight agitation, and the emitted fluorescence was examined with an EnSpire^®^ Multimode Plate Reader (Perkin Elmer, Madrid, Spain) at 570/585 nm.

### 4.5. Fluorescent Staining of Actin Distribution

For direct fluorescent staining, samples were treated first with the IC_90_ of the statin. After 24 h of incubation, cells were fixed with formaldehyde and deposited on a pre-coated coverslip. Later, cells were treated with Triton (0.1%) for 30 min followed by phalloidin-tetramethylrhodamine B isothiocyanate (phalloidin-TRITC; Sigma-Aldrich, Madrid, Spain) for another 30 min at room temperature. Finally, cells were washed with PBS. Cells were examined by Z-stack imaging with a 100× objective of EVOS FL Cell Imaging System AMF4300, Life Technologies, Carlsbad, CA, USA, at λexc = 540 nm and λem = 570 nm. As a positive control of actin depolymerization, methyl-β-cyclodextrin (BioReagent, Sigma-Aldrich, Madrid, Spain) was used [22]. Non-treated cells, containing 0.1% of DMSO, were considered as the negative control.

### 4.6. Evaluation of Programmed Cell Death (PDC) Induction

In all the fluorescence microscopy assays, the images were captured using an EVOS FL Cell Imaging System EVOS™ M5000 (100× magnification). In addition, the images shown are representative of the cell populations observed in each experimental condition. All the experiments were performed in triplicate.

### 4.7. Double-Stain Assay for Programmed Cell Death Determination

A double-stain apoptosis detection kit, Chromatin Condensation/Dead Cell Apoptosis Kit with Hoechst 33,342 and PI (Invitrogen, Madrid, Spain), and an EVOS FL Cell Imaging System EVOS™ M5000 (Life Technologies, Madrid, Spain), were used. The experiment was carried out by following the manufacturer’s recommendations, and 2 × 10^5^ cells/well were incubated for 24 h with the previously calculated IC_90_. The double-staining pattern allows for the identification of three groups in a cellular population; live cells show only a low level of fluorescence, cells undergoing PCD show a higher level of blue fluorescence (as chromatin condenses), and dead cells show low blue and high red fluorescence (as the propidium iodide stain enters the nucleus) [23].

### 4.8. Measurement of ATP

ATP levels produced over time were measured using a CellTiter-Glo^®^ Luminescent Cell Viability Assay (Promega, Madrid, Spain). The effect of the drug on the ATP inhibition was evaluated by incubating 2 × 10^5^ of cells/mL with the previously calculated IC_90_ of the present solution [23].

### 4.9. Intracellular ROS Production Using CellROX^®^ Deep Red Staining

The generation of intracellular ROS was detected using the CellROX^®^ Deep Red fluorescent probe (Invitrogen, Madrid, Spain). The cells were treated with the IC_90_ for 24 h and exposed to CellROX^®^ Deep Red (5 μM, 30 min) at 26 °C in the dark. Cells were observed with an EVOS FL Cell Imaging System EVOS™ M5000, Life Technologies, USA [24].

### 4.10. Analysis of Mitochondrial Membrane Potential

The collapse of an electrochemical gradient across the mitochondrial membrane over time was detected with the JC-1 Mitochondrial Membrane Potential Assay Kit (Cayman, Madrid, Spain). After being treated with IC_90_ of statins for 24 h, the cells were centrifuged (800× *g* 10 min) and re-suspended in JC-1 buffer. After that, 50 µL of each treated culture was incubated with 5 µL of JC-1 at 26 °C for 30 min. Images were taken on an EVOS FL Cell Imaging System EVOS™ M5000, Life Technologies, USA. The staining pattern allows the identification of two groups in a cellular population; live cells show only red fluorescence, while cells with low mitochondrial potential (undergoing PCD) show a higher level of green and red fluorescence [24].

### 4.11. Statistical Analysis

All data are expressed as the mean ± standard deviation of at least three independent experiments. To highlight the effect of both statin and strain, a statistical comparison was conducted using two-way analysis of variance (ANOVA). All the analyses and the graphics were performed by GraphPad Prism version 9.0. Statistical significance was set at *p* < 0.05.

## Figures and Tables

**Table 1:** Amoebicidal activity of the three statins against both cysts and trophozoites of *Acanthamoeba* spp. IC_50_ (µM).

	*A. castellanii* Neff		*A. polyphaga*		*A. griffini*		*A. quina*	
	Trophozoite	Cyst	Trophozoite	Cyst	Trophozoite	Cyst	Trophozoite	Cyst
Cerivastatin	0.114 ± 0.050 ^Aa^	0.704 ± 0.129 ^Aa^	0.939 ± 0.102 ^Ca^	20.037 ± 0.042 ^Da^	0.384 ± 0.064 ^Ba^	2.108 ± 0.046 ^Ba^	0.274 ± 0.046 ^ABa^	5.531 ± 0.941 ^Ca^
Rosuvastatin	5.583 ± 0.750 ^Ab^	18.168 ± 2.064 ^Ab^	16.463 ± 0.939 ^Cb^	>100 ^Cc^	8.582 ± 1.200 ^Bb^	23.738 ± 3.853 ^Ab^	5.659 ± 1.117 ^Ab^	81.620 ± 4.964 ^Bb^
Pitavastatin	0.389 ± 0.047 ^ABa^	3.040 ± 0.249 ^Aa^	1.907 ± 0.364 ^Ca^	48.774 ± 3.031 ^Bb^	0.859 ± 0.093 ^Ba^	5.306 ± 0.068 ^Aa^	0.229 ± 0.035 ^Aa^	5.921 ± 0.159 ^Aa^

Means within strains with different lower-case letters (a–c) are significantly different (*p* < 0.05). Means within compounds with different Upper-case letters (A–D) are significantly different (*p* < 0.05).

**Table 2:** IC_90_ of three different statins against *Acanthamoeba* spp. (µM).

	*A. castellanii* Neff	*A. polyphaga*	*A. griffini*	*A. quina*
Cerivastatin	0.305 ± 0.166	2.010 ± 0.010	1.850 ± 0.280	0.901 ± 0.143
Rosuvastatin	12.806 ± 2.081	47.728 ± 5.362	26.552 ± 5.583	14.010 ± 2.025
Pitavastatin	0.859 ± 0.163	5.208 ± 1.106	6.817 ± 4.728	0.695 ± 0.075

## References

[1] Martin-Navarro C.M., Lopez-Arencibia A., Sifaoui I., Reyes-Batlle M., Valladares B., Martinez-Carretero E., Pinero J.E., Maciver S.K., Lorenzo-Morales J. (2015). Statins and voriconazole induce programmed cell death in Acanthamoeba castellanii. Antimicrob. Agents Chemother..

[2] Thomson S., Rice C.A., Zhang T., Edrada-Ebel R., Henriquez F.L., Roberts C.W. (2017). Characterisation of sterol biosynthesis and validation of 14alpha-demethylase as a drug target in Acanthamoeba. Sci. Rep..

[3] Putaporntip C., Kuamsab N., Nuprasert W., Rojrung R., Pattanawong U., Tia T., Yanmanee S., Jongwutiwes S. (2021). Analysis of Acanthamoeba genotypes from public freshwater sources in Thailand reveals a new genotype, T23 Acanthamoeba bangkokensis sp. nov. Sci. Rep..

[4] Smith F.R., Korn E.D. (1968). 7-Dehydrostigmasterol and ergosterol: The major sterols of an amoeba. J. Lipid Res..

[5] Martin-Navarro C.M., Lorenzo-Morales J., Machin R.P., Lopez-Arencibia A., Garcia-Castellano J.M., De Fuentes I., Loftus B., Maciver S.K., Valladares B., Pinero J.E. (2013). Inhibition of 3-Hydroxy-3-Methylglutaryl–Coenzyme A Reductase and Application of Statins as a Novel Effective Therapeutic Approach against Acanthamoeba Infections. Antimicrob. Agents Chemother..

[6] Dudley R., Alsam S., Khan N. (2007). Cellulose biosynthesis pathway is a potential target in the improved treatment of Acanthamoeba keratitis. Appl. Microbiol. Biotechnol..

[7] Wang Y., Ren F., Song Z., Chen P., Liu S., Ouyang L. (2019). Statin use and the risk of ovarian and endometrial cancers: A meta-analysis. BMC Cancer.

[8] Maji D., Shaikh S., Solanki D., Gaurav K. (2013). Safety of statins. Indian J. Endocrinol. Metab..

[9] Hahn H.J., Abagyan R., Podust L.M., Roy S., Ali I.K.M., Debnath A. (2020). HMG-CoA Reductase Inhibitors as Drug Leads against Naegleria fowleri. ACS Chem. Neurosci..

[10] Pozo M., de Nicolas R., Egido J., Gonzalez-Cabrero J. (2006). Simvastatin inhibits the migration and adhesion of monocytic cells and disorganizes the cytoskeleton of activated endothelial cells. Eur. J. Pharmacol..

[11] Koyuturk M., Ersoz M., Altiok N. (2004). Simvastatin induces proliferation inhibition and apoptosis in C6 glioma cells via c-jun N-terminal kinase. Neurosci. Lett..

[12] Rizo-Liendo A., Sifaoui I., Arberas-Jimenez I., Reyes-Batlle M., Pinero J.E., Lorenzo-Morales J. (2020). Fluvastatin and atorvastatin induce programmed cell death in the brain eating amoeba Naegleria fowleri. Biomed. Pharmacother..

[13] de Souza W., Rodrigues J.C. (2009). Sterol Biosynthesis Pathway as Target for Anti-trypanosomatid Drugs. Interdiscip. Perspect. Infect. Dis..

[14] Kuipers H.F., Rappert A.A., Mommaas A.M., van Haastert E.S., van der Valk P., Boddeke H.W., Biber K.P., van den Elsen P.J. (2006). Simvastatin affects cell motility and actin cytoskeleton distribution of microglia. Glia.

[15] Chappell J., Wolf F., Proulx J., Cuellar R., Saunders C. (1995). Is the Reaction Catalyzed by 3-Hydroxy-3-Methylglutaryl Coenzyme A Reductase a Rate-Limiting Step for Isoprenoid Biosynthesis in Plants?. Plant Physiol..

[16] Copaja M., Venegas D., Aranguiz P., Canales J., Vivar R., Avalos Y., Garcia L., Chiong M., Olmedo I., Catalan M. (2012). Simvastatin disrupts cytoskeleton and decreases cardiac fibroblast adhesion, migration and viability. Toxicology.

[17] Sokalska A., Piotrowski P.C., Rzepczynska I.J., Cress A., Duleba A.J. (2010). Statins inhibit growth of human theca-interstitial cells in PCOS and non-PCOS tissues independently of cholesterol availability. J. Clin. Endocrinol. Metab..

[18] Tsubaki M., Takeda T., Kino T., Obata N., Itoh T., Imano M., Mashimo K., Fujiwara D., Sakaguchi K., Satou T. (2015). Statins improve survival by inhibiting spontaneous metastasis and tumor growth in a mouse melanoma model. Am. J. Cancer Res..

[19] Lee N., Tilija Pun N., Jang W.J., Bae J.W., Jeong C.H. (2020). Pitavastatin induces apoptosis in oral squamous cell carcinoma through activation of FOXO3a. J. Cell Mol. Med..

[20] Martin-Navarro C.M., Lorenzo-Morales J., Cabrera-Serra M.G., Rancel F., Coronado-Alvarez N.M., Pinero J.E., Valladares B. (2008). The potential pathogenicity of chlorhexidine-sensitive Acanthamoeba strains isolated from contact lens cases from asymptomatic individuals in Tenerife, Canary Islands, Spain. J. Med. Microbiol..

[21] Lorenzo-Morales J., Kliescikova J., Martinez-Carretero E., De Pablos L.M., Profotova B., Nohynkova E., Osuna A., Valladares B. (2008). Glycogen phosphorylase in Acanthamoeba spp.: Determining the role of the enzyme during the encystment process using RNA interference. Eukaryot. Cell.

[22] Zidovetzki R., Levitan I. (2007). Use of cyclodextrins to manipulate plasma membrane cholesterol content: Evidence, misconceptions and control strategies. Biochim. Biophys Acta.

[23] Sifaoui I., Reyes-Batlle M., Lopez-Arencibia A., Chiboub O., Rodriguez-Martin J., Rocha-Cabrera P., Valladares B., Pinero J.E., Lorenzo-Morales J. (2018). Toxic effects of selected proprietary dry eye drops on Acanthamoeba. Sci. Rep..

[24] Rodriguez-Exposito R.L., Nocchi N., Reyes-Batlle M., Sifaoui I., Suarez-Gomez B., Diaz-Marrero A.R., Souto M.L., Pinero J.E., Fernandez J.J., Lorenzo-Morales J. (2021). Antiamoebic effects of sesquiterpene lactones isolated from the zoanthid Palythoa aff. clavata. Bioorg. Chem..

